# Science and impact: the challenge faced by Dental Press Journal of Orthodontics

**DOI:** 10.1590/2177-6709.21.4.012-013.edt

**Published:** 2016

**Authors:** David Normando

**Affiliations:** 1 Adjunct professor, Universidade Federal do Pará (UFPA), School of Dentistry, Belém, Pará, Brazil. Coordinator, Universidade Federal do Pará (UFPA), Graduate program in Dentistry, and ABO-Pará, Specialization course in Orthodontics, Belém, Pará, Brazil.

*"In the middle of the road there was a stone.*
*There was a stone in the middle of the road."*

*Carlos Drummond de Andrade (Brazilian poet)*

Every year, in June, the major databases publish the impact exerted by scientific periodicals in the scientific context. While Thompson Reuters quantifies it as the journal's *impact factor*, SCImago database terms it *cites per doc*. However, both estimate the average number of citations of articles published in a certain periodical. The methods are very similar, with the only difference being the number of periodicals indexed in each database. 

Recently, in June 2016, data on the citations made in 2015 were published. The number of citations made in 2015 for articles published in 2013 and 2014 was divided by the total number of articles published in 2013 and 2014. Therefore, an impact factor of 2, for a certain periodical, means that, for every article published in 2013 and 2014, there was, on average, two citations of such articles in 2015. 

Despite criticism received by such bibliometrics, those evaluation systems have been widely used to assess the importance of a given periodical within its field of expertise. Overall, the more often a recently published article is cited, the greater its impact in the scientific context. Although it has expanded, Dentistry is not among the most impacting fields. While our best-ranked dental periodicals are near the average of having five citations per article, in Medical Sciences and Physics, a few periodicals have exceeded the number of 40 citations per article published. In Dentistry itself, specialties are heterogeneous. On one hand, our Periodontal periodicals have an average *cites per doc* of 5; on the other hand, in Orthodontics, *American Journal of Orthodontics and Dentofacial Orthopedics*; *Orthodontics and Craniofacial Research* and *Angle Orthodontists* presented *cites per doc* varying between 1.66 and 1.62, according to data provided in 2015.

**Figure 1:**
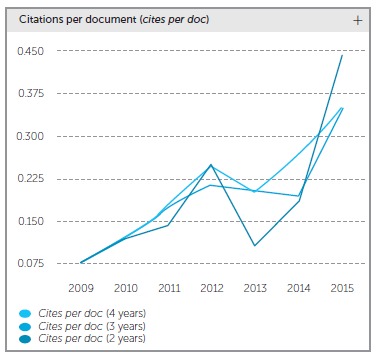
Dental Press Journal of Orthodontics cites per doc between 2009 and 2015.

Dental Press Journal of Orthodontics (DPJO), indexed in SCImago Scopus database, received its first *cites per doc* in 2009, when the journal was published in Portuguese only. As from 2010, DPJO articles began to be published in English. Since then, our impact index kept increasing and reached 0.25 in 2012 (Fig 1). At that point, there was an avalanche of good publications, and, in 2013, a setback was rendered necessary, as a consequence of having to double the number of articles published, so as to speed the publication process up.^1^ Such change increased significantly this equation denominator. After all necessary adjustments were made, in 2014, DPJO recovered part of its *cites per doc* loss from 2013. 

**Figure 2:**
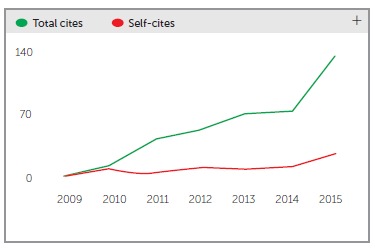
Dental Press Journal of Orthodontics total number of citations (total cites) and self-citations (self-cites) between 2009 and 2015.

Last June, we received data on the citations made in 2015, and found out we had an index equal to 0.44, a threefold increase in comparison to the previous year. There are two main reasons to warrant such significant growth. The first is the adjustment made to publication flow, reducing the time interval between manuscript submission and publication, and, most of all, having a smaller number of more selective articles published: while in 2013, 138 articles were published, in 2014 and 2015, 101 and 92 articles were published, respectively. Thus, we have been reducing the equation denominator. The second reason was having DPJO indexed in PubMed database in 2013, thus rendering knowledge published by this periodical more accessible. This indexation increased the chances of citation, thereby also increasing the equation numerator. Therefore, we went from 54 citations in 2012, before being indexed in PubMed, to 130 citations in 2015 (Fig 2). 

We have grown significantly; however, a lot more is yet to be reached. For the index to be determined in 2017, based on data from 2016, the equation denominator (number of articles published in 2014 and 2015) will be decreased by 20%. Additionally, it is hoped that the number of citations will increase, especially due to the periodical being indexed in PubMed Central. This database provides full, free access to all articles published from 2014 onwards, on the following website: www.ncbi.nlm.nih.gov/pmc/journals/2644. If we consider a potential decrease of 20% in the equation denominator and an increase of 80% in the equation numerator, we believe that we will be able to double DPJO's *cites per doc* in 2017 and reach a number close to 1.0. Should this be fulfilled, DPJO will become one of the six periodicals exerting the greatest impact in world-wide Orthodontics. If there once were so many stones in the middle of the road, many of them have already been removed, while some are yet to be. 

David Normando - editor-in-chief (davidnormando@hotmail.com)
